# Climatic and cultural changes in the west Congo Basin forests over the past 5000 years

**DOI:** 10.1098/rstb.2012.0304

**Published:** 2013-09-05

**Authors:** Richard Oslisly, Lee White, Ilham Bentaleb, Charly Favier, Michel Fontugne, Jean-François Gillet, David Sebag

**Affiliations:** 1 Institut de Recherche pour le Développement, UMR 208 IRD/MNHN, Patrimoines Locaux, Agence Nationale des Parcs Nationaux, BP 20379 Libreville, Gabon; 2 Agence Nationale des Parcs Nationaux, BP 20379 Libreville, Gabon; 3 Institut de Recherche en Ecologie Tropicale, BP 13354 Libreville, Gabon; 4 University of Stirling, Stirling FK9 4LA, UK; 5 Université de Montpellier 2, CNRS, ISEM, Place Eugene Bataillon, 34095 Montpellier, France; 6 Laboratoire des Sciences du Climat et de l'Environnement, UMR 8212 CNRS/CEA/UVSQ, Domaine du CNRS, 91198 Gif-sur-Yvette Cedex, France; 7 Université de Liège, Gembloux Agro-Bio Tech, Laboratoire de foresterie des régions tropicales et subtropicales, Passage des Déportés 2, 5030 Gembloux, Belgium; 8 Institut de Recherche pour le Développement, HydroSciences Montpellier, Université de Ngaoundéré, BP 454, Cameroon

**Keywords:** Holocene, archaeology, Congo–Ogooué basin, palaeoenvironment, climatic change, vegetation

## Abstract

Central Africa includes the world's second largest rainforest block. The ecology of the region remains poorly understood, as does its vegetation and archaeological history. However, over the past 20 years, multidisciplinary scientific programmes have enhanced knowledge of old human presence and palaeoenvironments in the forestry block of Central Africa. This first regional synthesis documents significant cultural changes over the past five millennia and describes how they are linked to climate. It is now well documented that climatic conditions in the African tropics underwent significant changes throughout this period and here we demonstrate that corresponding shifts in human demography have had a strong influence on the forests. The most influential event was the decline of the strong African monsoon in the Late Holocene, resulting in serious disturbance of the forest block around 3500 BP. During the same period, populations from the north settled in the forest zone; they mastered new technologies such as pottery and fabrication of polished stone tools, and seem to have practised agriculture. The opening up of forests from 2500 BP favoured the arrival of metallurgist populations that impacted the forest. During this long period (2500–1400 BP), a remarkable increase of archaeological sites is an indication of a demographic explosion of metallurgist populations. Paradoxically, we have found evidence of pearl millet (*Pennisetum glaucum*) cultivation in the forest around 2200 BP, implying a more arid context. While Early Iron Age sites (prior to 1400 BP) and recent pre-colonial sites (two to eight centuries BP) are abundant, the period between 1600 and 1000 BP is characterized by a sharp decrease in human settlements, with a population crash between 1300 and 1000 BP over a large part of Central Africa. It is only in the eleventh century that new populations of metallurgists settled into the forest block. In this paper, we analyse the spatial and temporal distribution of 328 archaeological sites that have been reliably radiocarbon dated. The results allow us to piece together changes in the relationships between human populations and the environments in which they lived. On this basis, we discuss interactions between humans, climate and vegetation during the past five millennia and the implications of the absence of people from the landscape over three centuries. We go on to discuss modern vegetation patterns and African forest conservation in the light of these events.

## 1. Introduction

Geological and biological studies carried out over the past 40 years have dispelled the illusion of the ‘eternal rainforest’ in equatorial Africa. Indeed, it is clear that during the last glacial maximum severe climatic conditions restricted dense equatorial forests in Africa into a small number of larger refuge areas and a complex mosaic of ‘micro-refugia’, where favourable conditions persisted [1–5].

The Holocene marked the return to milder conditions in Central and West Africa, and forests quickly regained lost ground, as evidenced by increasing levels of rainforest pollen in lake sediments across the region [6,7]. Several records from marine [8–10] and terrestrial climate archives [4,11] also suggest humid conditions interspersed by numerous climate fluctuations [12] during the Early and Middle Holocene followed by a dry Late Holocene.

**Figure 1.**
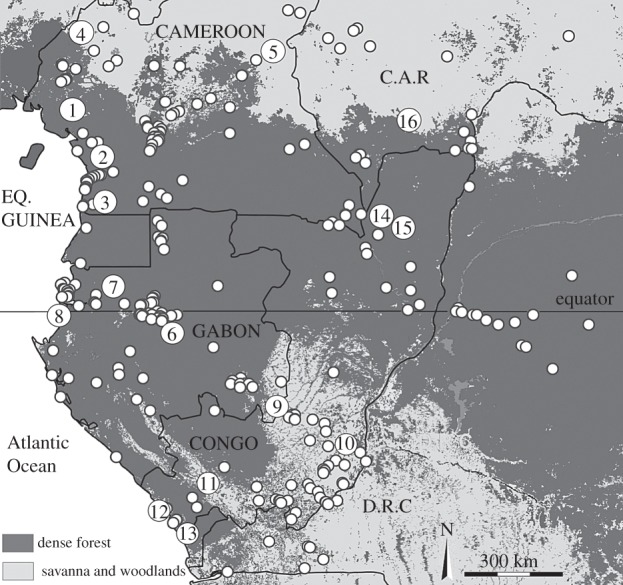
The study area (Central Atlantic Africa) showing the location of 328 archaeological sites and 16 lake coring sites in the dense forest and forest/savanna mosaic (references are cited in the main text): 1, Barombi Mbo; 2, Ossa; 3, Nyabesan; 4, Bambili; 5, Mbalang; 6, Kamalété; 7, Nguène; 8, Maridor; 9, Bilanko; 10, Ngamaka; 11, Sinnda; 12, Kitina; 13, Coraf/Songolo; 14, Mopo Bai; 15, Goualougo; 16, Bodingué.

In Central Africa, there is strong evidence for a significant forest regression event between 3000 and 2000 years BP. The Warm African monsoon declined from about 3500 years BP, causing a serious disturbance of the forest massif, as attested by many palynological and geological studies undertaken on sedimentary deposits of lakes (figure 1) in the Congo basin [3,13–27].

Pollen profiles show that humid forest trees were replaced by light-demanding pioneer and herbaceous species, characteristic of degraded forests and savannas between 3000 and 2500 BP [21].

The fragmentation of the Congolese forests, at a time when there were variations in the levels of many lakes in the region and corresponding changes of average surface water temperatures in the Gulf of Guinea [10], has been interpreted as a response to a generalized arid period in Central Africa [14,28], related to a weakening of the Atlantic monsoon. The palynological and geochemical data and diatoms at the site of Mbalang in Cameroon [22,29] demonstrate significant environmental change from 3200 years BP, linked to different hydrological conditions.

The consensus that has developed is that climatic conditions with a long dry season combined with more severe storm events resulted in severe erosion. A recent study [30] suggested that this can be explained by anthropogenic soil erosion, but others [31,32] argue that climate was the main driver. A large-scale spread of Bantu iron workers occurred only around 1900–1700 BP. It seems likely therefore that the first Bantu farmers opportunistically followed forest fragmentation to penetrate the forest zone.

These studies also demonstrate that the medieval period in the Northern Hemisphere (1100–800 years BP) is characterized by decadal fluctuations in the lake levels in Atlantic Central Africa [33], coinciding with the opening up of the canopy of mature forests in peripheral areas adjoining the forest block [14,20] as well as forest recovery in the central Gabon [34]. During the Little Ice Age (500–200 years BP), lake levels were low [16,33], cover of rainforests decreased and there was a change in the type of vegetation from evergreen to deciduous forests [16].

Archaeological studies have demonstrated that cultural and technological evolution occurred in parallel to these regional environmental changes. Here, we consider the distribution of archaeological sites discovered over the past 30 years in Cameroon and especially Gabon to evaluate the interactions between humans and the environment. Following a number of pioneering studies in the 1960s [35,36], systematic surveys were undertaken between 1980 and 1990. These early studies identified the major stages of cultural change in the region [37]. Subsequent studies in the period 1990–2000 focused on a number of major sites, providing a regional chronological reference for cultural changes [38–40]. More recently, the focus has been on interactions between man and the environment, focusing particularly on the impact of man on his habitat and the impacts of climate change on human societies [41–43]. In this synthesis, we assemble a database of 328 archaeological sites known in West Central Africa and analyse their distribution, in an attempt to better understand the relationships between climate, human demography and forest distribution through the Holocene.

## 2. Study area and methods

This is the first study to compile data on all known archaeological sites in the Central African rainforest region. The area under study includes six countries of Atlantic Central Africa (the southern half of Cameroon, mainland Equatorial Guinea (the island of Bioko has a different history), Gabon, Republic of Congo, the western part of the Democratic Republic of Congo and the southwest of the Republic of Central Africa). We divide the region into two generic biomes: forest and savanna/savanna–forest mosaic (figure 1).

We identified 328 archaeological sites with at least one reliable carbon date from the last 5000 years. Of these, 32% (106 of 328) were sites where one of the authors had been involved in research, including 20 sites for which no data have previously been published; the remainder were from the literature [38,40,43–91]. Oslisly *et al.* [92] provide additional details of dating methods, materials and precision.

We compiled all known published and unpublished radiocarbon dates for the 328 archaeological sites [92]. Of a total of 733 dates, 16 were rejected by researchers as being spurious, either owing to contamination or sampling error, 25 were modern, 94 were considered duplicates (very similar dates in the same site) and 12 were from Bioko island [93] (Equatorial Guinea), where there is an unbroken culture of pottery and polished stone tools from 700 AD until the arrival of Europeans in the eighteenth century. Dates on the same site a century or more apart were retained. A total of 586 dates were retained for this analysis, from a total of 328 sites.

Our method assumes that relative population numbers are related to the number of radiocarbon dates. However, caution is necessary, because sampling remains patchy reflecting the concentration of research activity and possible impacts of prevailing environmental conditions on charcoal preservation. It is also possible that the economic pursuits of pre-historic populations leave differential amounts of dateable material (i.e. iron-working versus mobile hunter–gatherers).

## 3. Results

**Figure 2.**
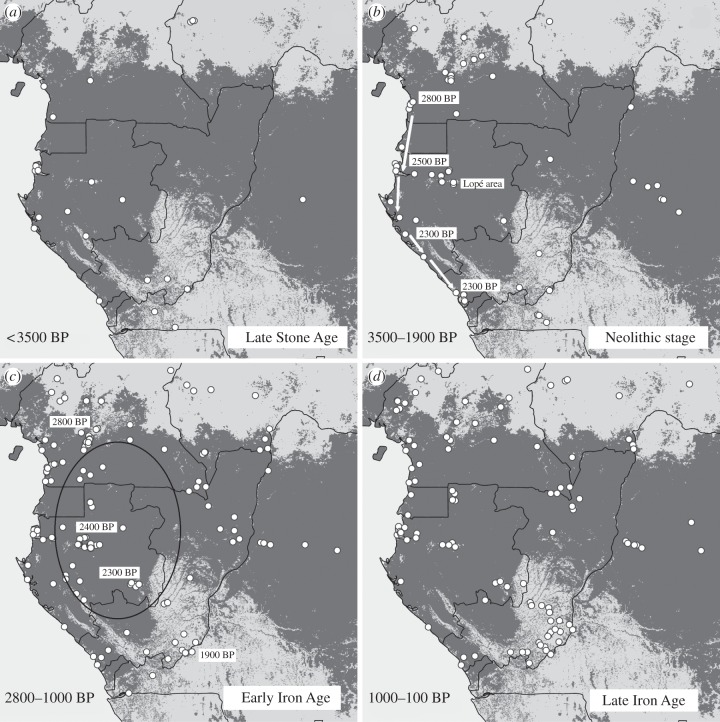
Location of 328 archaeological sites in Atlantic Central Africa for (*a*) the Late Stone Age, (*b*) the Neolithic stage, (*c*) the Early and (*d*) the Late Iron Ages.

Figure 2 shows the distribution of archaeological sites across the region divided between four cultural sequences: Late Stone Age; Neolithic stage; Early Iron Age and Late Iron Age.

Two-thirds of the sites are located in the modern forest/savanna mosaic. These areas are easier to prospect and archaeological sites often show up as sites with active erosion. Forest sites were generally discovered in places where artificial openings were made during construction of roads, railways, pipelines, dams and power plants, or during mining and forestry exploitation. Given the state of research today, it is not possible to say whether distribution of sites reflects the actual spatial distribution of archaeological sites or is simply an effect of patchy sampling.

**Figure 3.**
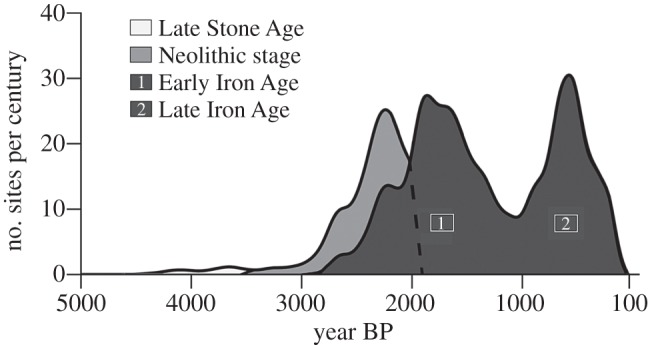
Graph of 586 radiocarbon dates for Atlantic Central Africa, distinguished on the basis of the character of associated archaeological remains.

Of 586 radiocarbon dates analysed, 33 were associated with Late Stone Age sites, 97 with Neolithic sites and 456 were Iron Age remains. The distribution of 586 carbon dates from 328 archaeological sites across the region is plotted by number of sites per century in figure 3. Figure 3 shows that few sites have been found dating prior to 3000 years BP. The Late Stone Age in the region ends around 3500 years BP. Neolithic sites increase in numbers from about 3000 years BP onwards, peaking at 2300 BP and petering out at 1900 years BP (except in Bioko island, which never had an Iron Age).

Map (*a*) shows the disparate nature of sites at the end of the Late Stone Age.

Map (*b*) shows the migration of the Malongo peoples from Cameroon to Congo over a period of 700 years.

Map (*c*) shows a north–south spread of iron smelting with older dates in the north and more recent dates in the south. The oval represents the region with the most iron-rich deposits.

Map (*d*) shows a gradual repopulation after the crash.

3500 years ago, a radical change is observed as groups of stone working hunter–gatherers give way across the region to new migrants, who settle the land and begin the first forms of rudimentary slash and burn agriculture [94]. Probably originating from northern Sahelian areas, these populations settled firstly along the edge of the forest block and benefited from forest fragmentation to penetrate further. They created small villages on hilltops and dug rubbish pits close to their houses, unlike Late Stone Age peoples who left their waste on the surface [39]*.* They mastered new technologies such as pottery and stone polishing and seem to have practised early forms of agriculture, as shown by the presence of stone hoes. This period, known as the ‘Neolithic stage’, occurs in the forest zone between 3500 and 2000 BP. People settle on hilltops and in dominant positions close to rivers.

**Figure 4.**
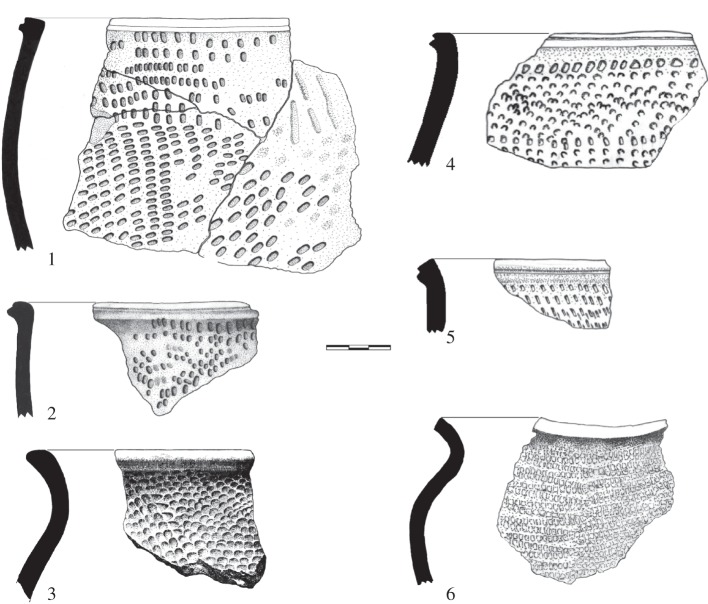
Typical Malongo tradition pottery: profile and vase fragments of fluted edges decorated with collars and swivel impressions. 1 and 2, Malongo (Cameroon); 3, Kogo (Equatorial Guinea); 4 and 5, Okala (Libreville) and 6, Tassi (Loango National Park) in Gabon (scale bar, 3 cm).

Beginning approximately 3200 years BP, a migratory wave travelled south from Cameroon along the Atlantic coast, taking advantage of the presence of a continuous string of narrow savannas that range from Equatorial Guinea to the mouth of the Congo. This tradition, defined by a common ‘Malongo’ pottery style, lasts from 2700 to 2000 years BP [75]. Their pottery containers were decorated with large zigzag patterns produced by a series of pivoting combs (figure 4). The edges of the vases are generally straight, thickened externally with a fluted lip with settings spanning from the neck to the base. The first Malongo pottery was dated at 2700 years BP at Bissiang [63] (figure 2). Homogeneous pottery remains found in the coastal areas of Kribi in Cameroon (2600–2000 BP) [75], near Kogo in Equatorial Guinea [95], at Libreville (2400–2000 years BP) [43], at Iguela and Mayumba in Gabon (2300–2100 years BP) and at Tchissanga in the Republic of Congo (2500–2000 years BP) [53,96] indicate that this was one cultural group that migrated southwards from Cameroon.

A second much more important current begins approximately 2800–2500 years BP in the hinterlands, and corresponds to the Early Iron Age. The first signs of iron smelting date back to 2800 years BP, at Oliga, in southern Cameroon [61]. The number of Iron Age sites rises rapidly from 2600 years BP onwards, peaking 1900 years BP. From 1600 to 1000 years BP, there is a drastic drop, suggesting that Atlantic Central Africa was almost devoid of people during this period.

Figure 2*c* shows the spread of iron smelting to the south over a period of 900 years. Note that the dates on the map indicate an initial spread from north to south in inland areas where geological formations rich in iron occur; and a second phase along the coast, indicating it was principally a result of commercial exchanges, although some iron furnaces have been found.

These metal workers demonstrated a very good knowledge of geological formations and established their settlements on hilltops [41].

They made a range of forms and decorative styles of ceramics that differ markedly from those of the Neolithic cultures, suggesting that they replaced or displaced peoples. Bi-lobed and careened pots decorated using comb swivels disappear in favour of generalized closed vessels with open edges, with more intricate decorations: concentric circles and bands of incised parallel lines mainly in the upper part of the body, where handles and gripping appendices were fitted.

In the middle valley of the Ogooué, corresponding rock engravings are found that include comparable geometric shapes with those found on the pottery as well as animal figures [39]. Oslisly [41] has mapped the southwards spread of characteristic pottery styles associated with these populations, demonstrating their gradual migration to the south. The distinct lack of any hybrid forms and decorations suggests that the populations that moved in displaced those already there.

Increasing densities of archaeological sites around 2000–1900 years BP demonstrate a demographic explosion of metal working populations (figures 2*c* and 3). Using iron tools, these peoples had the potential to profoundly affect the forest by slash and burn forms of agriculture and it is likely that they also managed forest–savanna mosaics using fire as a hunting strategy. They also produced great quantities of charcoal during iron reduction operations and would have maintained savannas through the lighting of bush fires [74,97].

In addition to evidence of gathering of rainforest fruits and seeds, such as *Antrocaryon klaineanum, Canarium schweinfurthii* and *Coula edulis* [41,42], there is also evidence of extensive use of oil palm beginning from 3000 years BP in the Yaoundé area [94] and at 2800 years BP at Otoumbi in central Gabon [38]. There is also an isolated record around 2200 BP of cultivation of pearl millet, *P. glaucum,* in the modern forest zone of southern Cameroon [98]. This observation is consistent with suggestions that it was generally drier at this time, with longer, more pronounced dry seasons.

**Figure 5.**
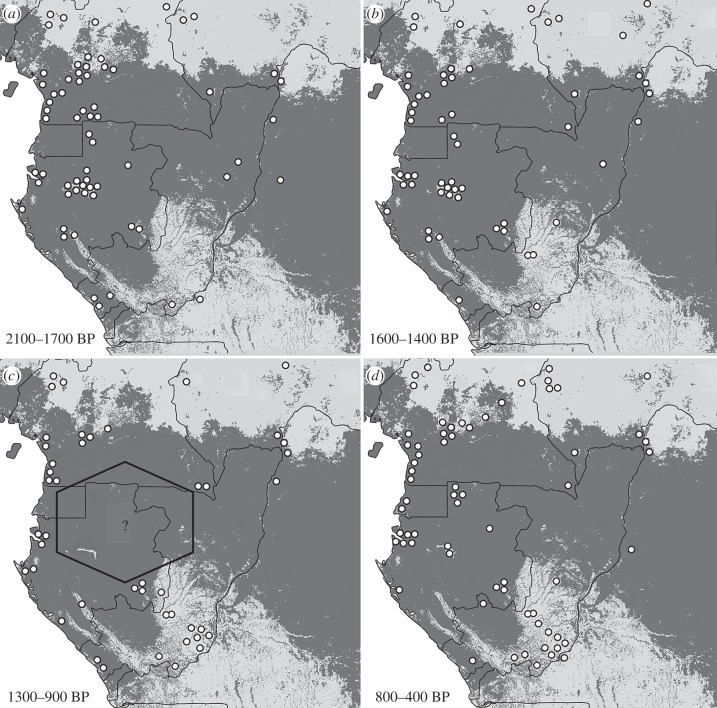
Maps showing distribution of dated archaeological sites in the periods: (*a*) 2100–1700 years BP; (*b*) 1600–1400 years BP; (*c*) 1300–900 years BP; (*d*) 800–400 years BP. The relations between these populations are described in detail in [39].

Figure 5 presents four maps showing the distribution of archaeological sites in four periods:

Map (*a*): the period from 2100 to 1700 years BP indicates that there were many sites of Iron Age and Neolithic stage in Cameroon and Gabon.

Map (*b*): the period from 1600 to 1400 years BP shows a slight iron age population deflation.

Map (*c*): the period from 1300 to 900 years BP reveals a total absence of dates in the forest hinterland South Cameroon/Centre and North of Gabon/North of Congo and little human presence in the peripheral areas of the forestry block. There was a clearly defined ‘population crash’ from 1300 to 900 years BP in the central Gabon [41].

Map (*d*): the period from 800 to 400 years BP shows that the forest block has been recolonized by Late Iron Age populations.

From 1000 years BP, new populations of metalworkers settled in the region, achieving their greatest numbers around 500 BP, in the pre-colonial period. The division in terms of abundance of sites is corroborated in some regions by cultural differences. For example, in central Gabon, iron ore reduction structures built above ground with clay in the Early Iron Age disappear in favour of pits, and the ceramic traditions are totally different, later styles being characterized by frequent use of recurrent patterns created using wooden roulettes, with no evidence of cultural exchange [41].

The metallurgists of the Late Iron Age resettled along hilltops and practised shifting cultivation [39]. Ceramics of this period include large and small pots of flattened spherical shape with out-curved apexes and pots of uneven curvature, as well as clay pipes indicating smoking of tobacco. The decoration of these pots is unique, with small circular motifs made with knotted strips of plant material forming herringbone patterns. The designs on pots form a band of variable width high on the sphere, and similar patterns are found on the clay bowls of pipes.

The maximum expansion of the new metallurgists peaks around 500 years BP (figure 3) and then decreases to around 100 years BP. While the end of this last phase coincides with the end of the Little Ice Age, other factors are probably important. This period was marked by the first contacts with Europeans, which caused a radical shift in Aboriginal culture: the traditional pottery was supplanted by colonial pots. The ‘cultural shift’ is not the only impact: these contacts also saw the development of the slave trade, which had a profound impact on populations. It is very difficult to assess the impacts of the slave trade, because data are scarce and difficult to verify. Some data exist for Sao Tomé and for the Kongo Empire but few are available for Gabon. Picard-Tortorici & François [99] put forwards a figure of 18 000 slaves leaving the Gabonese coast, which had an estimated population of 100 000. This suggests that the consequences on human population densities may have been significant. Slave trails penetrated far into the interior, reaching Lopé in the Central Gabon [100] and further southeast. Data on vegetation composition and structure in Lopé have allowed for modelling of vegetation change over the past 2000 years, indicating that a spurt of savanna colonization corresponds to the period covered by the slave trade [101]. It seems likely that depopulation in the Lopé region resulted in reduced frequencies of savanna fires, allowing forest expansion, and reduced intensity of agriculture, resulting in forest succession from young to more mature formations.

However, the subject has received little attention and deserves further study by archaeologists, historians and geographers, although, from about 500 years BP onwards, the archaeological data are of limited value owing to the fact that archaeologists rarely radiocarbon date sites that they know on the basis of stratigraphy and pottery to belong to the relatively recent past. Hence, for this period, we have to rely on historical documents or undertake new detailed archaeological surveys.

## 4. Discussion and conclusion

In the early part of the Holocene Stone Age, human settlements seem to be few and far between in the forest belt of West Central Africa and there is little to suggest that man played an overly determinant role in the ecosystem. The Late Stone Age, which started at about 40 000 years BP [102], ended approximately 3500 years BP, when Neolithic cultures migrated from Cameroon into Gabon and Congo along the coast. These peoples were the first to make pottery, which would have greatly increased their ability to store and preserve foodstuffs, and their polished stone hoes indicate an early form of agriculture.

With the arrival of iron smelting and working, which moved into the region from the Sahel and central Sahara around 2800 years BP, human impacts on the rainforest would have increased significantly. During the same period, approximately 3000–2000 years BP, Central Africa was also affected by a worsening climate that contributed to the fragmentation of the forest block and probably also to the accelerated expansion of Bantu peoples towards the south.

The increase in numbers of archaeological sites from 2000 years BP through to about 1600 years BP (figure 3) suggests that human population numbers increased greatly through this period. Despite somewhat fragmented evidence it seems safe to conclude that the reduction of forest cover at this time, through a combination of changes in the hydrological cycle and increased anthropogenic pressure, may have resulted in a deforestation peak on a scale similar to that which has affected West Central Africa over the last century or so [103].

To date, there is little evidence of what was being cultivated. De Maret records oil palm, *Elaeis guineensis*, for the first time in the region 3000 years BP in the Yaoundé area [94] and Oslisly [38] and Fay [104] record it, respectively, 2850 years BP in the Lopé region of Central Gabon and from 2400 years BP in the Nouabalé Ndoki region of northern Congo. In some areas of coastal Cameroon, there is evidence of extensive oil palm groves [75], and extensive forest fires in the valley of the Lélédi and Offoué in the Central Gabon at around 1800 years BP [97] are consistent with agriculture on a comparable scale with that recorded in Okomu in southwest Nigeria around 700 years BP, continuous over at least 1000 km^2^ [105–107].

It will require further archaeological exploration across Central Africa in order to fully document and understand the implications of pre-historic man on vegetation patterns across the African rainforest zone, but recent studies in Central Africa as well as other studies further afield [108] suggest that humanity's impact in the recent past may have been significant. Indeed, the region known as the Sangha interval, a low biodiversity corridor separating richer forests of the Ogooué and Congo basins [109], has been revealed in recent years to contain numerous archaeological sites, as well as vast deposits of oil palm seeds dating to the period between 2400 and 1000 BP (peaking at 1700 BP). The forests in this region remain dominated by species indicative of old secondary vegetation (*Entandrophragma*, *Triplochiton*, etc.) as well as extensive formations with a dense understorey dominated by species of Marantaceae, indicative of cultivation, savanna colonization or forest fires [104,110,111], and it is possible that their low diversity is a result of severe disturbance by humans over the past three millennia, particularly considering that their actions have been coupled with phases of climate stress.

The decrease in abundance between 1600 and 900 years BP and possible disappearance of humans from large parts of the landscape between 1350 and 900 years BP would have had an equally marked, if different, impact on vegetation (figure 5*c*). Palaeoenvironmental data reveal that from 1400 years BP a stronger monsoon would have favoured forest regeneration [21], so reduced frequencies of anthropic fires at this time would have created conditions favourable to wide-scale forest regeneration [74,97].

It is interesting to hypothesize about the cause of the human population decline. If the population rise was built on cultivation of pearl millet (*P. glaucum*) [98] during a period of climatic stress for rainforest vegetation and from 1400 years BP conditions became too humid for this species, then it would have been difficult to maintain high populations, and people may have migrated away in search of a climate appropriate for millet cultivation. If Mbida's report of banana cultivation [112] in the Yaoundé area proves reliable this hypothesis would seem less likely, because bananas would have thrived in the new climate, but if no alternative was available, then this hypothesis seems plausible.

An alternative hypothesis, which remains impossible to test due to the fact that acidic soils preclude the discovery of human bodies dating to this time, is that the risk of epidemics would have increased as the human population density increased. Old literature on epidemics in Atlantic Central Africa shows that sleeping sickness seems to have been the disease that most affected the populations of the forest, owing to its high mortality rate. At the end of the nineteenth century, the French colonial administration reported a major outbreak of trypanosomiasis, which resulted in the disappearance of populations in eastern Gabon and northern Congo [113]. Around the year 1920, when Dr Eugene Jamot fought against human African trypanosomiasis in South Cameroon, he found that 116 000 persons out of 664 000 examined (17%, with peaks at 30% in some places) were infected with sleeping sickness, a deadly disease with no traditional cure [114].

Recently, apes that had risen to unusually high densities in old secondary vegetation in northeast Gabon and northwest Congo following displacements of human populations died of Ebola in density-dependent epidemics [115] that might mirror what happened to people some 1400 years BP.

Another possibility is that the extreme weather events of AD 535–536, known to be the most severe and protracted short-term episodes of cooling in the Northern Hemisphere in the past 2000 years [116], had an impact in Central Africa. The event is thought to have been caused by an extensive atmospheric dust veil, possibly resulting from a large volcanic eruption in the tropics [117]. Its effects were widespread, causing unseasonal weather, crop failures and famines worldwide.

These hypotheses can be confirmed or refuted only by further research. While we attempt to demonstrate that the number of radiocarbon dates is related to demographic changes, other questions related to shifts in human activities across the landscape require further studies.

Irrespective of the cause, the evidence for the population decline seems sound, and the implications of the rise and subsequent fall of human populations for vegetation are likely to be significant. In more recent times, the impacts of the slave trade and the subsequent forced relocation of rural populations to roads and urban centres by colonial authorities, leaving vast areas in Gabon and Congo devoid of people [118], also had significant impacts on vegetation, such as the distribution of Okoumé trees (*Aucoumea klaineana*) in Gabon [101].

The pattern that emerges over the past 5000 years is a complex interaction of variations in climate and in human population density, distribution and ability to impact on forest vegetation. While it is clear from botanical work that these changes have not overwhelmed the signature of longer-term climate change resulting in the cyclical retraction of forests into refuges and expansion across the Congo basin [5,119], no scientific study of vegetation, including work on the dynamics of carbon stocks, should ignore the possibility of disturbance linked to human activities over the past two millennia having a significant bearing on the results. Archaeologists have developed methods of mapping past human activity using trees such as *A. klaineana*, *Lophira alata* and *Baillonella toxisperma*, all of which are associated with old village sites [120]. Furthermore, many of the forests with the highest densities of large mammals, which tend to be prioritized for conservation, are in areas that have been significantly impacted by humans over the past 1000 years or so [103].

This synthesis of 30 years of archaeological studies allows us to describe the main stages in cultural development, as well as key changes in climatic conditions through the past 5000 years. The picture we have painted is not without analogy to the current situation, where human populations are growing in the context of increasing climatic stress. Currently, it seems unlikely that we will avoid the large-scale deforestation that such circumstances have caused repeatedly in the past. The combination of climate change and increased logging pressure is likely to result in increased frequencies of forest fires [110], whereas the global appetite for productive agricultural land is likely to see more and more of the Central African forests converted to large-scale oil palm plantations and other commercial crops [121]. The REDD+ process seemed for a while to offer some potential for preservation of extensive tracts of forests, but interest in this process seems to be stagnating, with very few concrete success stories to bolster flagging enthusiasm of political leaders.

The archaeological data presented in this paper suggest that several waves of forest disturbance and oil palm cultivation have affected the Congo Basin over the past three millennia and that the forest has been relatively resilient. Were the conditions to be put in place to favour forest growth, it is likely that regeneration would be rapid. In the meantime, conservationists should look to mirror the patterns of forest survival that have made it possible for forests to recover quickly once given the chance.
